# Comparative Analysis of Tongue Indices between Patients with and without a Self-Reported Yin Deficiency: A Cross-Sectional Study

**DOI:** 10.1155/2017/1279052

**Published:** 2017-05-17

**Authors:** Su-Ryun Kim, Woojin Choi, Inkwon Yeo, Dong-Hyun Nam

**Affiliations:** ^1^Department of Biofunctional Medicine and Diagnosis, College of Korean Medicine, Sangji University, Wonju 26382, Republic of Korea; ^2^Department of Neuropsychiatry, College of Korean Medicine, Sangji University, Wonju 26382, Republic of Korea; ^3^Department of Statistics, Sookmyung Women's University, Seoul 04310, Republic of Korea

## Abstract

We investigated the hypothesis that Yin-deficient patients have a reddened tongue with less coating. We screened 189 participants aged 20 to 49 years, complaining of headache. To classify patients in terms of Yin deficiency, we used two self-reporting Yin-deficiency questionnaires (Yin-Deficiency Questionnaire and Yin-Deficiency Scale) and diagnosis by a doctor. Based on the tests, a total of 33 subjects were assigned to a Yin-deficient group and 33 subjects were assigned to a nondeficient control group. Tongue images were acquired using a computerized tongue diagnostic system, for evaluating tongue indices. The tongue coating percentage and tongue redness were calculated as the mean *a*^⁎^ value of both the whole tongue area (WT *a*^⁎^) and the tongue body area (TB *a*^⁎^). The tongue coating percentage of the Yin-deficient group (34.79 ± 10.76) was lower than that of the nondeficient group (44.13 ± 14.08). The WT *a*^⁎^ value of the Yin-deficient group (19.39 ± 1.52) was significantly higher than that of the nondeficient group (18.21 ± 2.06). However, the difference in the TB *a*^⁎^ value between the two groups was not significant. In conclusion, we verified that Yin-deficient patients had less tongue coating and tended to have a more reddish tongue than nondeficient patients.

## 1. Introduction

In traditional Korean medicine (TKM) and traditional Chinese medicine (TCM), pattern identification is used to determine the pathological state of a patient. The patterns, called Zheng, imply the cause, nature, location, and the severity of the disease. Thus, a traditional medicine practitioner gathers and analyzes the symptoms and signs present in a patient to determine the course of action for treating an underlying disease. To identify such patterns, practitioners typically perform inspections that involve listening, smelling, inquiry, and palpation [1].

Inspection of the tongue is an important diagnostic approach in pattern identification in TKM because the tongue is considered to reflect the status of the human body, such as that of the qi-blood and the fluid of the internal organs, rapidly [2]. The practitioner examines the color, shape, moisture, and movement of the tongue body (which is a composite of muscles and vessels) and the color, thickness, and distribution of the tongue coating (a fur-like attachment to the dorsum of the tongue).

A Yin-deficiency (YD) pattern is a pathological state. The clinical symptoms of YD are typically associated with emaciation, tidal fever, night sweats, malar flush, palpitations, insomnia, dizziness, tinnitus, dry mouth and throat, constipation, a reddened tongue with little coating, and a fine rapid pulse [3]. In particular, a reddened tongue with little coating is regarded as a distinctive sign of YD. A “reddened tongue” sign indicates that the color of the tongue is reddish rather than the normal pale red color of the tongue, and a tongue with “little coating” indicates that either no or only a small amount of tongue coating is visible on the tongue body.

However, whether the sign of a reddened tongue with little coating is associated with YD in patients remains unverified. Moreover, the definition of a “reddened tongue” has not been definitively established. A “reddened tongue” can be interpreted as the result of less coating on the tongue body, which reveals a pinker or redder color, or may reflect increased blood flow.

Several TKM and TCM studies [4–7] have investigated the tongue status of Yin-deficient patients. In those studies, the Yin-deficient patients were mainly grouped at the discretion of the practitioners; this methodology lacked objectivity. Thus, the results of these studies are not highly reliable. For objective assessment of a Yin-deficient status, several tools have been developed, such as the Yin-Deficiency Questionnaire (YDQ), the Yin-Deficiency Scale (YDS), Shi-pattern analysis, the Yin/Yang-deficiency Questionnaire, and the Yin-Xu Body Constitution Questionnaire [8]. Nevertheless, most of these tools do not include a tongue inspection or rely only on the subjective tongue inspection of the practitioner [9–11]. Some studies [12–15] have reported that inspection of the tongue has a low reliability, and thus an objective approach to inspection is needed. Therefore, several types of quantitative tongue assessment systems, such as a computerized tongue diagnostic system (CTDS), have been developed to solve this problem. Han et al. [16] investigated the relationship between tongue status and YD with the YDQ, using a CTDS; the results of this study were not consistent with the traditional medicine theory of tongue diagnosis. However, a single investigation is not sufficient to gain an understanding of the relationship between tongue status and YD.

Therefore, the present study quantitatively investigated the hypothesis that YD in patients is associated with a reddened tongue with little coating. We compared evaluation indices of the tongue between a Yin-deficient group of patients and a nondeficient control group. To identify patients with a YD and nondeficient patients, we used two self-reporting questionnaires, the YDS and YDQ [1, 17], both of which have been previously validated. To investigate differences in the tongue status between the two patient groups, we evaluated indices of the amount of tongue coating and the tongue color using a CTDS.

## 2. Materials and Methods 

### 2.1. Ethics Approval and Consent to Participate

This study was conducted according to the standards of the International Committee on Harmonization of Good Clinical Practice and the revised version of the Declaration of Helsinki. Written informed consent was obtained from all of the study participants before the experiment. The Institutional Review Board of the Traditional Korean Medicine Hospital of Sangji University in Wonju, Republic of Korea, approved the experimental protocol (IRB number SJ IRB-Human-15-010).

### 2.2. Study Design

Between December 2015 and August 2016, a total of 189 people were screened from among the outpatients complaining of headache at the Traditional Korean Medicine Hospital of Sangji University in Wonju, Republic of Korea. All patients were aged between 20 and 49 years. The exclusion criteria were pregnancy, severe systemic organ diseases, use of drugs within the 7 days prior to screening, smoking, vitamin B use, or an abnormal condition of the tongue, that is, the inability to open the jaw and protrude the tongue stably, the presence of a geographic tongue, bleeding, malformation of the tongue, or the presence of dental braces. Thirty-one patients were excluded in the process of screening according to these criteria.

**Figure 1:**
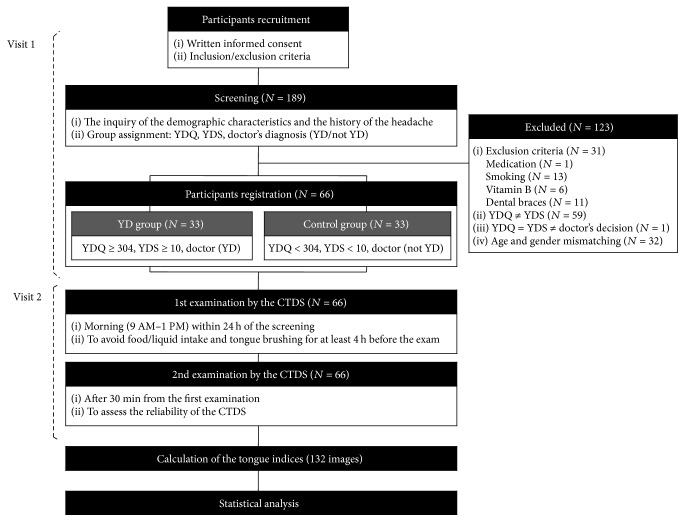
Flow chart of the experimental design of the present study. YDS: Yin-Deficiency Scale; YDQ: Yin-Deficiency Questionnaire; YD: Yin deficiency; CTDS: computerized tongue diagnostic system.

Demographic characteristics were recorded for each patient. In addition, the duration of the headache was recorded and severity of the headache was also assessed using a visual analog scale. After an experimenter explained the YDS and YDQ, each patient completed the questionnaires. To prevent any bias due to a misunderstanding of the questionnaires, a TKM doctor with more than 10 years of clinical experience diagnosed each patient as Yin-deficient or nondeficient, while blinded to the results of the questionnaires and without inspection of the patient's tongue. The diagnosis of a YD or a nondeficient status in patients was thus based on the scores of the YDS and the YDQ as well as the independent opinion of the doctor. Fifty-nine patients were excluded because of discordance between the two questionnaires. One YD subject was also excluded from our study based on the decision of the doctor that he was nondeficient. Then, the nondeficient patient group was matched to the group of patients who had YD, in terms of age and gender. Thirty-two nondeficient individuals were also excluded for matching. Finally, 66 participants were registered for this study. The overall design of the study is presented in Figure 1.

### 2.3. Tongue Examination

All subjects had their tongues examined using a CTDS. The subjects were required to avoid food and liquid intake for at least 4 h before the tongue examination and to refrain from brushing their teeth and tongue. In consideration of the influence of circadian rhythms, the tongue examination was conducted in the morning within 24 h of the screening. To image the tongue, the patients were instructed on how to touch their faces and protrude their tongues to the CTDS. After the patient exercised the procedure several times, opening their mouths and extruding the tongue to reveal their entire tongue, the CTDS was used to acquire an image of the tongue. To assess the reliability of the CTDS, an image of the tongue was acquired again in the same manner after 30 min. After image acquisition, we assessed whether the patients experienced any adverse event. The initial 66 images that were acquired during this examination were analyzed in a comparison of the YD patient group and the nondeficient patient group. The second group of images was only used to calculate the reliability of the CTDS.

### 2.4. The Yin-Deficiency Questionnaire and the Yin-Deficiency Scale

In 2007, the YDQ was developed for objective measurement of the severity of a YD [1]. The reliability and validity of the YDQ have been previously established [18], and the YDQ has been used to investigate the relationship between a YD and other symptoms, such as dry mouth, hot flush, and skin disorders [19–21]. The YDQ is comprised of 10 questions to which the patients responded by marking the severity of their symptoms on a 100 mm bar. The cut-off score for a YD diagnosis is 304 [22].

Park et al. [17] developed the YDS, which suggests the optimum cut-off score for a diagnosis of YD as 10. The YDS includes a total of 27 questions to which the patients responded on a scale of 1 to 7, to indicate the severity of a YD. To discriminate patients with YD, the score of the YDS was calculated on a dichotomous scale; the scores of 1, 2, 3, and 4 were converted to 0 points and the scores of 5, 6, and 7 were converted to 1 point.

### 2.5. Image Acquisition System

A CTDS (CTS-1000, Daiseung Medics, Seoul, South Korea) was used to acquire tongue images. The CTDS comprised a camera, illuminators, an external light shielding system, and analysis software. The camera was set up using a color board (Color Checker Passport, X-Rite, Grand Rapids, MI, USA) before the capture of tongue images. The operator captured an image of the tongue together with two 18% gray chips (R27, Kodak, Rochester, NY, USA) on the bilateral sides of the tongue, to evaluate the gray balance and perform fast color adjustments. Additional details of the procedure for image acquisition using the CTDS are described in a previous study [23].

### 2.6. Calculation of the Tongue Indices

**Figure 2:**
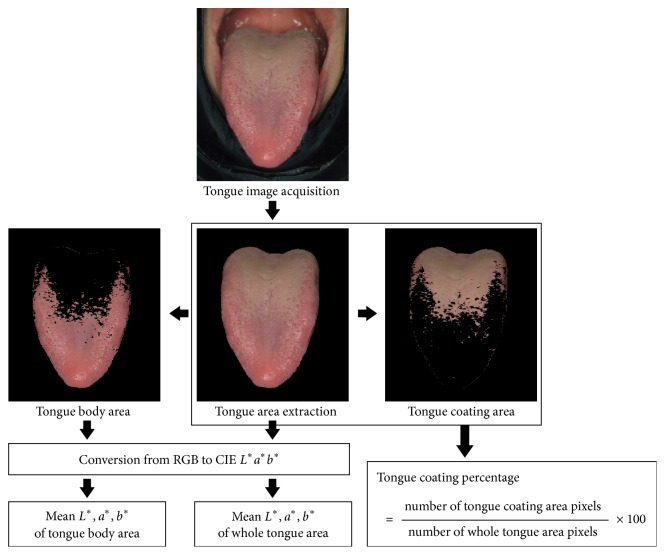
Calculation of tongue indices. The tongue area was extracted from an acquired tongue image. The tongue coating area was distinguished from the tongue body area based on the difference in color. The RGB color values in the areas of interest were converted into CIE-*L*^*∗*^*a*^*∗*^*b*^*∗*^ color values. Then, the mean *L*^*∗*^*a*^*∗*^*b*^*∗*^ values of whole tongue area and tongue body area were calculated. The tongue coating percentage was calculated as the percentage of the pixel number of the tongue coating area to the pixel numbers of the whole tongue area.

The region of the tongue was extracted from the captured image. The tongue coating area was distinguished from the tongue body area based on the difference between the *a*^*∗*^ value for the tongue body and tongue coating. The RGB color values of each pixel in the areas were converted to CIE-*L*^*∗*^*a*^*∗*^*b*^*∗*^ color values. Then, the mean values of *L*^*∗*^*a*^*∗*^*b*^*∗*^ for the whole tongue (WT) area and tongue body (TB) area were calculated. The tongue coating percentage was calculated as the percentage of the pixel number of the tongue coating area to the pixel number of the whole tongue area. The process for calculating these tongue indices is shown in Figure 2. The tongue coating percentage was used as the index to estimate the amount of tongue coating. The redness of the tongue was estimated as the mean *a*^*∗*^ value for the whole tongue area (WT *a*^*∗*^) and the mean *a*^*∗*^ value for the tongue body area (TB *a*^*∗*^).

### 2.7. Statistical Analysis

The baseline characteristics of the participants are presented using descriptive statistics. Differences in the demographic characteristics and the severity of the headache between the YD group and nondeficient control group were compared using a chi-squared test for categorical data and an independent samples* t*-test for continuous data. If the assumption of normality was not confirmed, Mann–Whitney *U* test was used. To evaluate the homogeneity of the headache duration, we used the proportional odds model [24]. Levene's test was performed to assess the equality of variances for the demographic characteristics and the tongue indices between the two groups. Intraclass correlation coefficients (ICCs) were calculated to assess the reliability of the CTDS. ICC values above 0.8 indicate acceptable reliability according to Shrout and Fleiss [25]. A binary logistic regression analysis was performed to predict the probability of YD occurrence according to the indices. Statistical analyses were performed using IBM SPSS Statistics 23 (IBM Corporation, Armonk, NY, USA) or SAS version 9.4 (SAS Institute Inc., Cary, NC, USA). A* p* value < 0.05 was considered statistically significant.

## 3. Results

### 3.1. Baseline Characteristics of the Participants

The baseline characteristics of the participants are shown in Table 1. The demographic differences between the YD group and nondeficient control group were not significant. In addition, differences in headache duration between the two groups were not significant; the estimated coefficient of the group effect was 0.484 (standard error = 0.45) and the corresponding *p* value was 0.280. However, the headache severity of the YD group was significantly higher than that of the nondeficient group.

### 3.2. Tongue Indices

**Figure 3:**
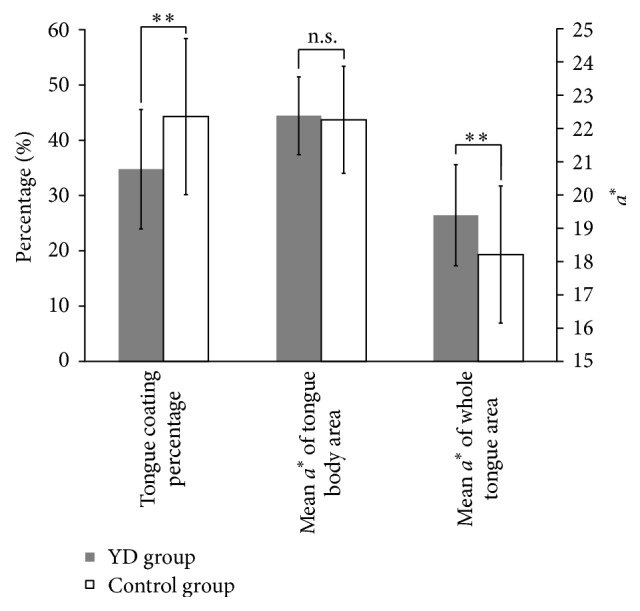
Comparison of the tongue indices between the Yin-deficient group and the nondeficient control group. The tongue coating percentage of Yin-deficiency (YD) group (34.79%) was significantly lower than that of the control group (44.13%). The mean *a*^*∗*^ value of whole tongue area (WT *a*^*∗*^) was significantly different between the two groups; the WT *a*^*∗*^ value of the Yin-deficient group (19.39 ± 1.52) was higher than that of the control group (18.21 ± 2.06). However, the mean *a*^*∗*^ value of tongue body was not significantly different between the two groups. Vertical bars: ±SD; ^*∗∗*^*p* < 0.01; n.s.: not significant.

The results for all of the tongue indices between the YD group and nondeficient control group are presented in Table 2. The tongue coating percentage of the YD group (34.79 ± 10.76) was significantly lower than that of the nondeficient group (44.13 ± 14.08; *p* = 0.004). The WT *a*^*∗*^ value was significantly different between the two groups (*p* = 0.010); the WT *a*^*∗*^ value of the YD group (19.39 ± 1.52) was higher than that of the nondeficient group (18.21 ± 2.06). However, there was no significant difference in the TB *a*^*∗*^ values between the two groups (*p* = 0.729). The differences in the tongue indices are presented in Table 2 and Figure 3.

### 3.3. The Reliability of the Computerized Tongue Diagnostic System

Independent examination of two images per participant revealed the acceptable reliability of the CTDS, as shown in Table 3. All the ICCs of the tongue coating percentage and the *L*^*∗*^*a*^*∗*^*b*^*∗*^ values of the tongue body and whole body exceeded 0.8 (tongue coating percentage = 0.826; the TB *a*^*∗*^ value = 0.801; the WT *a*^*∗*^ value = 0.812).

### 3.4. Binary Logistic Regression

**Figure 4:**
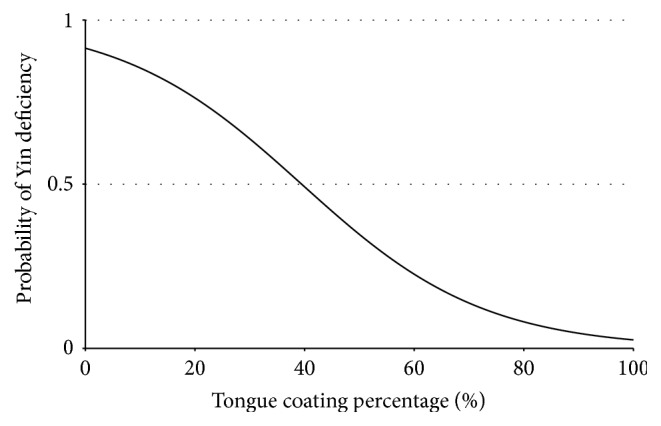
Logistic regression curve. Decrease of tongue coating percentage indicates an increased probability of Yin deficiency. [Probability of Yin deficiency = exp(0.06*X* − 2.368)/(1 + exp(0.06*X* − 2.368)); *X* = tongue coating percentage (%)].

We conducted binary logistic regression for YD with the tongue coating percentage and the WT *a*^*∗*^ value.  Table 4 and Figure 4 show the regression model for the tongue coating percentage. The Wald test indicated that the tongue coating percentage was a significant predictor of YD (*p* = 0.007). In the regression model, the WT *a*^*∗*^ value was removed because it was not significant as a predictor of YD (*p* = 0.759) once the tongue coating percentage was included in the model. The effective coating percentage for which there will be a 50% chance of YD is 39.47% (2.368/0.06).

### 3.5. Adverse Event

None of the patients experienced any adverse events during the study period.

## 4. Discussion

**Figure 5:**
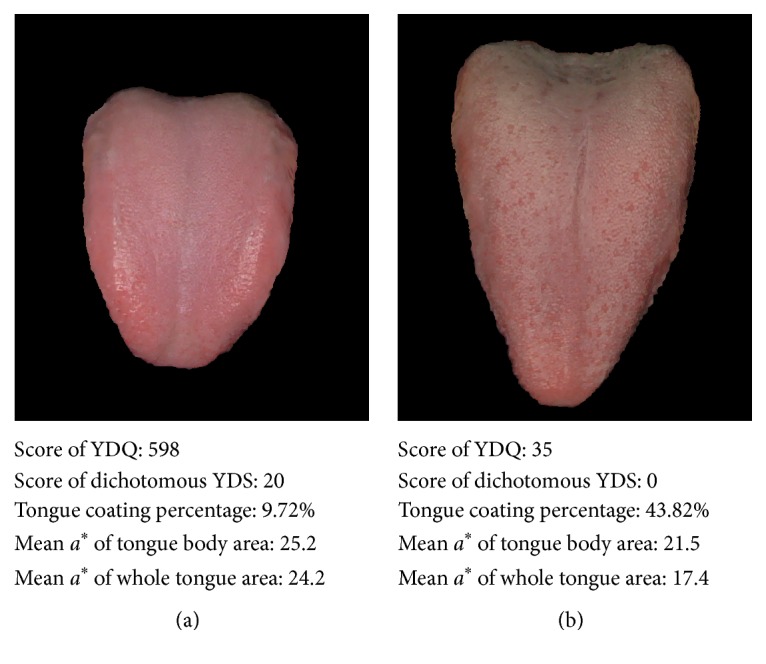
Representative tongue images from the Yin-deficient and control groups. A tongue coating percentage was used as an index to estimate the amount of tongue coating. The redness of the tongue was measured as the mean *a*^*∗*^ value of both the whole tongue area and the tongue body area that remained after excluding the tongue coating area. (a) A tongue image of a Yin-deficient patient is shown, and (b) a tongue image from a nondeficient control patient is shown. Note that these examples show that the tongue coating percentage of the Yin-deficient patient is lower and that the mean *a*^*∗*^ value of the whole tongue area is higher than those of the nondeficient patient. The mean *a*^*∗*^ value of the tongue body area showed a relatively small difference between the two samples.

The results of the present study showed that the tongue coating percentage of the YD group was lower and that the WT *a*^*∗*^ value was higher than those of the nondeficient control group. These results are consistent with the TKM tongue diagnosis theory that the tongue in patients with YD is reddened with little coating compared to the tongues of nondeficient individuals. However, we did not observe any significant difference in the TB *a*^*∗*^ values between the YD and control groups. This negative result suggests that a reddish color of the tongue is the result of a decrease in the tongue coating on the body of the tongue. Representative tongue image examples from the YD patient and the nondeficient control are shown in Figure 5.

Previous studies have found negative results when attempting to verify TKM and TCM theories. However, these negative results may have been due to the lack of objectivity in the methodology used in these studies; for most of these studies, the researchers subjectively evaluated the pattern and the tongue status, which likely biased their conclusions. In contrast, the present investigation used a more objective approach. We used two self-reporting questionnaires, in addition to the expert opinion of a practitioner to minimize the influence of experimenter bias, or the influence of an inaccurate self-report due to misunderstanding of the questionnaires. The patients that we identified as Yin-deficient complained of symptoms related to a YD; thus, our approach was likely objective and accurate.

Another strength of our study was the use of a CTDS to evaluate the tongue. With this approach, the conditions for obtaining tongue images remained constant. Moreover, our CTDS allowed a quantitative evaluation of tongue indices. The high ICC level demonstrates the reliability of our study.

In order to compare the tongue status with different patterns, the participants should have the same chief complaint but should be differentiated by variable patterns including YD. Therefore, we recruited patients who were all suffering from headache because the clinical pattern of a headache in TKM theory varies: wind-cold, wind-heat, wind-dampness, ascendant hyperactivity of the liver yang, static blood, heat syncope, dampness-phlegm, qi-deficiency, blood deficiency, and YD [26].

Previous studies have suggested that the amount of tongue coating increases with aging [27–30]; therefore, the participants in our study were limited to an age range of 20 to 49 years, and the control group was age-matched to the YD group. Consequently, the mean and standard deviation of age for both the YD group and the control group were similar. In addition, some studies have suggested that circadian rhythms influence oral status [31–33]; therefore, all of the examinations in our study were performed at approximately the same time in the morning. Moreover, our pilot study (not published) found that the correlation between the responses to the YD questionnaires and the tongue indices was weaker as time progressed after completing the questionnaires; therefore, we conducted our examination within 24 h of the screening.

We also performed Pearson's correlation coefficient analysis to investigate relationships among the tongue indices and the YDQ and YDS scores. We found that the tongue coating percentage and test scores for YD were negatively correlated. Moreover, the WT *a*^*∗*^ values were positively correlated with the test scores for YD. However, this correlation was weak, as the correlation coefficients were between 0.25 and 0.35. This result might be due to the lack of severity in the group of YD patients; the mean total YDQ scores and the dichotomized YDS score for the YD patients were not high (YDQ = 485.5 ± 94.4; YDS = 14.4 ± 2.9), considering that the highest scores achievable on these tests are 1,000 and 27, respectively. The WT *a*^*∗*^ values were very strongly correlated with the tongue coating percentage (*r* = −0.922, *p* < 0.001) and were not a significant predictor of YD when the tongue coating percentage was included in the logistic regression model. This implies that a “reddened tongue” can be interpreted as being the result of reduced coating on the tongue body, rather than the result of a reddened tongue body in the present study. Future studies should investigate patients with more severe YD to investigate whether a stronger correlation might appear.

The present study has several limitations. First, as mentioned above, the self-reporting screening could have been inaccurate due to the participant's misunderstanding of the questionnaire. Second, the number of participants that were included in our study was based on previous studies of tongue coating. Thus, our study design was more suitable for the investigation of tongue coating than of tongue color. Finally, although patterns of deficiency might be more easily observed in the elderly, the participants in the present study were relatively young, to increase the generalizability of the results.

## 5. Conclusions

Our results show that the tongue coating in self-reported YD patients tended to be decreased and the overall tongue color tended to be reddish compared to that in a nondeficient control group. These observations are consistent with traditional medicine theory of tongue diagnosis.

## Figures and Tables

**Table 1:** Baseline characteristics of the participants.

	Yin deficiency	Control	Total	*p* value
	(*N* = 33)	(*N* = 33)	(*N* = 66)	
Sex (M/F)^(a)^	10 (30.3)/23 (69.7)	12 (36.4)/21 (63.6)	22 (33.3)/44 (66.7)	0.602
Age (years)^(b)^	30.82 ± 7.49	30.82 ± 8.71	30.82 ± 8.06	1.000
Height (cm)^(c)^	165.99 ± 6.31	165.97 ± 9.27	165.98 ± 7.87	0.993
Weight (kg)^(b)^	63.67 ± 12.85	63.26 ± 12.52	63.46 ± 12.59	0.896
Systolic blood pressure (mmHg)^(b)^	107.61 ± 8.51	111.42 ± 13.90	109.52 ± 11.59	0.183
Diastolic blood pressure (mmHg)^(b)^	72.36 ± 6.67	74.30 ± 10.82	73.33 ± 8.97	0.385
Pulse rate (bpm)^(b)^	68.36 ± 10.98	66.58 ± 8.75	67.47 ± 9.89	0.467
Temperature (°C)^(b)^	36.46 ± 0.19	36.40 ± 0.18	36.43 ± 0.18	0.140
Headache duration				
Duration < 1 week	6 (18.2)	12 (36.4)	0.48 ± 0.45	0.280
1 week ≤ duration < 1 month	10 (30.3)	5 (15.2)		
1 month ≤ duration < 6 months	4 (12.1)	6 (18.2)		
6 months ≤ duration	13 (39.4)	10 (30.3)		
Headache VAS^(b)^	4.55 ± 1.86	3.42 ± 1.95	3.98 ± 1.97	0.020^*∗*^

Data are presented as *n* (%) or mean ± SD and were compared by chi-squared test^(a)^, independent samples *t*-test^(b)^, or Mann–Whitney *U* test^(c)^. Headache duration was compared by estimated coefficient of group effect and mean ± SE; ^*∗*^*p* < 0.05.

**Table 2:** Tongue indices.

Variables	Levene's test		Yin deficiency	Control	*p* value
	*F*	*p* value	(*N* = 33)	(*N* = 33)	
Tongue coating percentage (%)	3.195	0.079	34.79 ± 10.76	44.13 ± 14.08	0.004^*∗∗*^
Tongue body area					
*L*^*∗*^	1.352	0.249	51.72 ± 2.41	50.50 ± 2.83	0.064
*a*^*∗*^	0.892	0.349	22.38 ± 1.17	22.26 ± 1.61	0.729
*b*^*∗*^	0.209	0.649	10.77 ± 1.81	11.39 ± 2.02	0.194
Whole tongue area					
*L*^*∗*^	0.893	0.348	50.07 ± 2.23	49.51 ±2.79	0.373
*a*^*∗*^	1.937	0.169	19.39 ± 1.52	18.21 ± 2.06	0.010^*∗∗*^
*b*^*∗*^	0.107	0.745	10.30 ± 1.73	10.84 ± 2.00	0.244

The variables were compared by an independent samples *t*-test; ^*∗∗*^*p* < 0.01.

**Table 3:** The reliability of the computerized tongue diagnostic system.

	ICCs	Standard error (95% CI)
Tongue coating percentage	0.826	0.715–0.893
Tongue body area		
*L*^*∗*^	0.800	0.673–0.877
*a*^*∗*^	0.801	0.675–0.878
*b*^*∗*^	0.865	0.779–0.917
Whole tongue area		
*L*^*∗*^	0.805	0.682–0.881
*a*^*∗*^	0.812	0.694–0.885
*b*^*∗*^	0.876	0.797–0.924

**Table 4:** Binary logistic regression coefficients.

Model	*B*	SE	Wald	df	*p* value	exp(*B*)	95% CI for exp(*B*)	
							Lower	Upper
Tongue coating percentage	−0.060	0.022	7.354	1	0.007	0.941	0.901	0.983
Constant	2.368	0.903	6.885	1	0.009	10.678		

## Abbreviations

CTDS: Computerized tongue diagnostic system
TB: Tongue body area
TB *a*^*∗*^: The mean *a*^*∗*^ value for the tongue body area
TCM: Traditional Chinese medicine
TKM: Traditional Korean medicine
WT: Whole tongue area
WT *a*^*∗*^: The mean *a*^*∗*^ value for the whole tongue area
YD: Yin deficiency
YDS: Yin-Deficiency Scale
YDQ: Yin-Deficiency Questionnaire.
